# Antihypertensive Treatment and Renal Protection: the role of drugs inhibiting the renin-angiotensin-aldosterone system

**DOI:** 10.1007/s40292-013-0027-y

**Published:** 2013-10-04

**Authors:** Francesca Viazzi, Giovanna Leoncini, Roberto Pontremoli

**Affiliations:** Università degli Studi e I.R.C.C.S. Azienda Ospedaliera Universitaria San Martino-IST, Istituto Nazionale per la Ricerca sul Cancro, Viale Benedetto XV, 16125 Genoa, Italy

**Keywords:** Chronic kidney disease, Hypertension, Renin-angiotensin-aldosterone inhibitors, Proteinuria

## Abstract

The prevalence of chronic kidney disease, currently estimated to vary between 8 and 12 % in the general population, is steadily rising due to aging and to the ongoing epidemic of hypertension and type 2 diabetes. Even in its early stages, chronic kidney disease entails a greater risk for cardiovascular mortality, and its prevention and treatment is rapidly becoming a key medical issue for many health care systems worldwide. Adequate blood pressure control and reduction of urine protein excretion, preferably obtained by the use of renin-angiotensin-aldosterone system inhibitors, have traditionally been considered the mainstay of therapeutic strategies in patients with renal disease. Given the pivotal role of renin-angiotensin-aldosterone system activity in the pathogenesis and progression of renal and cardiovascular damage, a more profound inhibition of the system, either by the use of multiple agents or by a single agent at high dosage has recently been advocated, especially in the presence of proteinuria. Recent trials, however have failed to confirm the usefulness of this therapeutic approach, at least in unselected patients. This article will critically review the current literature and will discuss the clinical implications of targeting the renin-angiotensin-aldosterone system in order to provide the greatest renal protection.

## Introduction

Chronic kidney disease (CKD) is currently estimated to affect between 8 and 12 % of the general population, and its prevalence may be as high as 20–30 % in subgroups at increased risk such as patients with diabetes, hypertension and the elderly [1–3]. The economic impact of renal diseases is steadily growing worldwide and its prevention and treatment is becoming a priority for many public health systems. In its early stages, besides the obvious risk of progressing to end stage renal disease (ESRD) and the need for renal replacement therapy, renal damage represents a formidable multiplier of global cardiovascular (CV) risk [4–6]. In fact, the presence of increased albuminuria or a mild reduction in glomerular filtration rate, albeit typically asymptomatic and often overlooked, entails a manifold higher risk of unfavorable CV events [7]. CKD, especially in the presence of significant proteinuria, typically remains a progressive condition and since the decline in renal function is paralleled by further increase in the burden of risk, many patients actually die from CV complications well before reaching ESRD [8, 9]. Although optimal treatment for CKD has not yet been established, current therapeutic strategies aim at correcting and optimizing traditional and kidney specific CV risk factors. In this respect, high blood pressure may well be regarded as a special condition since it is known to be both a cause and a consequence of renal damage [10]. Effective antihypertensive treatment has clearly been shown to attenuate the worsening of renal function and to reduce proteinuria [11]. While optimal blood pressure levels are still a matter of debate and may vary on the basis of clinical conditions such as the presence of diabetes and/or the degree of proteinuria, there is consensus that maximizing the reduction of urinary protein excretion is associated with better renal outcome, regardless of blood pressure changes and may represent an intermediate target of treatment. International guidelines unanimously recommend the use of renin-angiotensin-aldosterone system (RAAS) inhibitors as the pharmacological agent of choice to treat hypertension and provide renal protection in CKD patients [12, 13]. The well established pathogenetic role of the RAAS in promoting and sustaining renal damage, both at experimental and clinical levels, has led researchers to test the hypothesis that a more profound inhibition of the system, achieved through the concomitant use of multiple agents or through the supramaximal dosage of a single agent may confer additional therapeutic benefits. Recent clinical trials however do not seem to confirm this somewhat oversimplified therapeutic scenario and once more emphasize the need for a careful and individualized approach to antihypertensive treatment in the renal patient [14, 15]. This article will briefly review the recent literature on the pros and cons of the use of RAAS-inhibitors and provide a practical algorithm to optimize antihypertensive treatment in patients with CKD.

## Blood Pressure Lowering Reduces Proteinuria and Conveys Renal and CV Protection

The magnitude of systolic blood pressure reduction as well as the degree of proteinuria reduction has been related to renal outcome in patients with non diabetic CKD, with greater renal protection being obtained when these two variables are lowered to a range of 110–130 mmHg and below 1.5 g/day, respectively [16–18]. Furthermore, results of the RENAAL and IDNT trials [19] indicate that residual blood pressure and urine protein excretion levels, i.e. those that can be achieved under optimal antihypertensive treatment, are far more important for long term renal protection as compared to baseline values. Specifically, the prognostic power of residual proteinuria seems to outweigh that of blood pressure since a graded relationship between the degree of proteinuria and the risk of reaching ESRD was observed for each systolic blood pressure strata [20]. Furthermore, better long-term renal survival in patients with overt proteinuria assigned to more intensive blood pressure reduction was confirmed by recently published data from the AASK study [21]. A similar relationship between albuminuria and renal outcome has been shown to apply also to earlier stages of the disease as indicated by an association of albuminuria reduction with better preservation of GFR in the STENO-2 study carried out on patients with type 2 diabetes [22]. Based on these data, international guidelines have rather unanimously recommended blood pressure values <130/80 mmHg (with some indicating even lower values, i.e. <125/75 mmHg when proteinuria exceeds 1 g/die) [23]. Recently, however, the wisdom and evidence in favor of such a strict, often difficult to achieve blood pressure target has been disputed since the untoward, paradoxical increase in mortality (so called J curve phenomenon) observed with progressively lower blood pressure regimens has been confirmed in several trials, especially in diabetics and coronary artery disease subgroups. Thus, it appears that while preservation of kidney function may well be better obtained by maximal blood pressure reduction, especially in the presence of proteinuria, an overly ambitious target may not always be recommended in the overall interest of at least some CKD patients, as global risk profile and the need to optimize cerebrovascular and cardiac protection must be taken into account as well [24].

## RAAS Inhibitors: Renal Protection Beyond Blood Pressure Reduction?

Extensive experimental and clinical evidence, accrued over more than three decades, document the deleterious effects of increased RAAS activity at the vascular and tissue level, especially within the kidney [25]. An excess of angiotensin II increases intraglomerular pressure by preferentially constricting the efferent arterioles, thus promoting glomerular hypertension and protein trafficking through the glomerular basal membrane [26, 27]. Furthermore, angiotensin II stimulates aldosterone production and triggers the activation of a cascade of profibrotic cytokines whose activation ultimately leads to cellular glomerular sclerosis and tubule-interstitial fibrosis [28, 29]. Given these pathophysiological premises it is certainly not surprising that antihypertensive agents which specifically inhibit RAAS activity at various levels may exert antiproteinuric and renoprotective effects even beyond their systemic hemodynamic effect on blood pressure. Clinical confirmation of this hypothesis was first provided in the early nineties by the Collaborative Study Group Trial, whose results documented how the ACE-Inhibitor captopril afforded greater proteinuria reduction and better renal survival as compared to placebo in type 1 diabetic patients with overt renal disease. Blood pressure reduction however was slightly but significantly lower in the captopril arm, making it harder to quantify precisely how much of the observed renal protection was specifically attributable to the drug’s mechanism of action [30]. Greater renal protection as compared to placebo was later reported with other ACE-Inhibitors (ACE-I), namely ramipril and benazepril, in the REIN study [31] and in the AIPRI study [32], both conducted on non diabetic patients with proteinuric kidney diseases.

At the beginning of this century, the then new class of angiotensin II type I receptor blockers (ARBs) was tested (again vs placebo) on the subgroup of patients that were rapidly becoming the most prevalent in renal practice everywhere, i.e. those with type 2 diabetes. In two similar landmark trials [33, 34], losartan and irbesartan, given on top of standard antihypertensive therapy, proved superior to placebo in reducing proteinuria and the rate of renal function loss over time, despite similar blood pressure reduction. Although only a few clinical trials have formally tested a head to head comparison between ACE-Is and ARBs, based on the results of several small studies and one reasonably large trial (comparing telmisartan and enalapril in patients with type 2 diabetes and incipient renal disease) [35], ACE-Is and ARBs have traditionally been assumed to provide comparable renal protection, in the context of undisputed superior tolerability of the latter. More recently, a large trial [14], albeit not specifically carried out on renal patients, reported similar renal protection with ramipril and telmisartan in high risk patients. Despite minimal blood pressure differences between treatment arms, which required statistical adjustment, several [17, 18] but not all meta-analyses [36] have sided with the notion of a certain degree of additional, blood pressure independent renal protective effect with ACE-I or ARBs. Guidelines have so far acknowledged these results indicating ARBs as the preferred choice in type 2 DM patients, and ACE-I for non diabetic renal patients.

## Optimizing RAAS Inhibition in Clinical Practice: The Need for Limitation

After the initial enthusiasm over the results of trials conducted with ACE-Is and ARBs, investigators realized that in the presence of proteinuria, a disappointingly large fraction of CKD patients, with or without diabetes, tend to progress toward ESRD over a few years, despite optimal antihypertensive treatment and RAAS-inhibition. In the meantime, new experimental data uncovered several biochemical mechanisms, such as non ACE-dependent Angiotensin II formation pathways [37] or counter-regulatory supramaximal Angiotensin II production in the presence of ATI1 type I receptor blockade [38, 39], the so called aldosterone escape phenomenon, which provided the basis for hypothesizing an incomplete blockade of the RAAS both under ACE-I or ARB treatment. This led investigators to search for and test new pharmacologic strategies to obtain more profound and complete inhibition of the RAAS in order to achieve greater renal protection (Table 1).

**Table 1:** Major clinical trials on new pharmacologic RAAS inhibition strategies to improve renal protection

Trial name	Publ. year	Drug	Comparator	Number of patients	Population	Renal end points	Mean follow-up	Results
**ACE-I plus ARB versus ACE-I or ARB**								
*Clinical trials*								
CALM	2000	Lisinopril 20 mg plus candesartan 16 mg	Lisinopril 20 mg or candesartan 16 mg	199	type 2 diabetes with microalbuminuria	Albuminuria reduction	12 weeks monotherapy with ARB or lisinopril followed by 12 weeks monotherapy or combination treatment	Significantly larger decrease of albuminuria and larger decrease in blood pressure under dual blockade
IMPROVE	2007	Ramipril 10 mg plus irbesartan 150–300 mg	Ramipril 10 mg	405	Hypertensive patients with albuminuria (100 %), diabetes or cardiovascular disease	Albuminuria reduction	20 weeks	Larger decrease of UAE and BP under dual blockade
ONTARGET	2008	Telmisartan 80 mg plus ramipril 10 mg	Telmisartan 80 mg or ramipril 10 mg	25,620	Patients with coronary, peripheral, or cerebrovascular disease or diabetes with end-organ damage	All-cause death, doubling serum creatinine, ESRD	4.7 years	The number of primary outcomes was similar for telmisartan and ramipril but was increased with combination therapy
ORIENT	2011	Olmesartan plus ACE-I	ACE-I	577	Patients with diabetic nephropathy and overt proteinuria secondary to type 2 diabetes	Doubling of serum creatinine, ESRD and death	3.2 years	Olmesartan did not improve primary composite renal endpoint on top of ACEI, while it significantly decreased blood pressure, proteinuria and rate of change of reciprocal serum creatinine
*Metanalyses*								
Kunz et al.	2008	ACE-I + ARB combination	ACE-I or ARB	1192	Patients with or without diabetes and with microalbuminuria or proteinuria	Proteinuria reduction	1–4 months 5–12 months	The ARB and ACE-I combination reduced proteinuria more effectively than either agent alone in the short term, while in the long term this was only true in comparison with ARB
Maione et al.	2011	ACE-I + ARB combination	ACE-I or ARB	5,442	Patients with albuminuria	ESRD and progression to macroalbuminuria	NA	Development of end-stage kidney disease and progression to macroalbuminuria were reduced significantly with ACEI versus placebo and ARB versus placebo but not with combination therapy versus monotherapy
Susantitaphong et al.	2013	ACEI + ARB; ACEI or ARB + ARA; ACEI + ARB + DRI; ACEI + ARB + ARA.	ACEI or ARB or ARA or DRI;	4,975	CKD patients	eGFR and albuminuria variations; doubling of the serum creatinine level, hyperkaliemia	NA	Combined RAAS blockade therapy was associated with a significant net decrease in glomerular filtration rate, albuminuria and proteinuria. Combined RAAS blockade therapy was associated with a 9.4 % higher rate of regression to normoalbuminuria and a 5 % higher rate of achieving the blood pressure goal. However, combined RAAS blockade therapy was associated with a significantly greater incidence of side effects
**DRI plus ACE-I or ARB**								
AVOID	2008	Aliskiren 150–300 mg plus losartan 100 mg	Losartan 100 mg (ARB)	599	Type 2 diabetes with nephropathy and hypertension	Albuminuria reduction	6 months	Under dual blockade larger decrease of albuminuria and blood pressure
ALTITUDE	2012	Aliskiren 150–300 mg on top of optimal treatment with an ACE-I or an ARB	Placebo	8,561	Type 2 diabetes at high cardiorenal risk	All-cause death, doubling serum creatinine, ESRD	Prematurely stopped after a median follow up of 33 months	Lack of benefit of hard endpoint and potentially harmful side-effects
**ARA plus ACE-I or ARB versus ACE-I or ACE-I plus ARB**								
*Clinical trials*								
Epstein et al.	2006	Eplerenone 50 or 100 mg plus enalapril 20 mg	Enalapril 20 mg	268	DM with macroalbuminuria	Albuminuria reduction and incidence of hyperkalemia	12 weeks	Larger decrease in albuminuria with dual blockade and similar decrease in blood pressure. No difference in hyperkaliemia
Mehdi et al	2009	Spironolactone 25 mg plus lisinopril 80 mg	Lisinopril 80 mg or Losartan 100 mg plus Lisinopril 80 mg	81	DM with macroalbuminuria	Albuminuria reduction	48 weeks	Larger decrease of albuminuria with MRI/ACE-I than with ACE-I. Similar decrease in albuminuria with ARB/ACE-I as with ACE-I
*Meta-analyses*								
Navaneethan et al.	2009	Spironolactone from 25 to 50 mg plus ACE-I or ARB Eplerenone from 50 to 200 mg + ACE-I	ACE-I or ARB	845	CKD with albuminuria	Albuminuria reduction, GFR decline	NA	Larger decrease in albuminuria and larger decrease in blood pressure under dual blockade. No difference in renal function decline. More gynecomastia and hyperkaliemia with spironolactone but not with eplerenone
Bomback et al.	2008	ARA	ARA on top of ACE-I or ARB	NA	Patients with proteinuric kidney disease	Changes in proteinuria, in blood pressure, and in glomerular filtration rate; rates of hyperkalemia	NA	Adding ARA to ACE-inhibitor and/or ARB therapy yields significant decreases in proteinuria without adverse effects of hyperkalemia and impaired renal function
**Supramaximal doses**								
*Clinical trials*								
DROP	2007	Valsartan 320, or 640 mg	Valsartan 160 mg	391	Hypertensive patients with type 2 diabetes mellitus and albuminuria	Albuminuria reduction, regression to normoalbuminuria	30 weeks	Higher valsartan doses were associated with greater reductions in mean albuminuria, independently of blood pressure reductions
SMART	2009	Candesartan mg or 128 mg	Candesartan 16 mg	269	CKD with persistent proteinuria despite candesartan 16 mg	Proteinuria reduction	30 weeks	Candesartan 128 mg/d results in a further significant reduction in urinary protein excretion independently of blood pressure control. Reductions in blood pressure were not different across the three treatment groups.
ROAD	2007	Individual uptitration of benazepril (median 20 mg/day; range 10–40) or individual uptitration of losartan (median 100 mg/day; range 50–200)	Benazepril 10 mg/die or Losartan 50 mg/die	360	Non diabetic, proteinuric CKD	Doubling of the serum creatinine, ESRD, or death	3.7 years	Higher dosages of ACEI or ARB at comparable blood pressure control led to a reduction of proteinuria and of renal end points

*ACE-I* angiotensin converting enzyme inhibitors, *ARA* aldosterone receptor antagonist, *ARB* angiotensin II receptor blockers, *CKD* chronic kidney disease, *DRI* direct renin inhibitor, *ESRD* end-stage renal disease, *GFR* glomerular filtration rate, *UAER* urinary albumin excretion rate

### Dual RAAS Inhibition

Preliminary results of several small studies and three meta-analyses [40–42] suggest that greater reduction of proteinuria could be obtained by combining treatment with an ACE-I and an ARB. One small study conducted on patients with type 1 diabetes, with a triple cross over design in 20 diabetic patients with overt proteinuria demonstrated that high dose losartan or its association with lisinopril (both at recommended doses) were superior to recommended doses of losartan in reducing proteinuria [43]. These data contributed to raising expectations that dual RAAS blockade could translate into long term reduction of hard renal endpoints. However, following formal retraction of the COOPERATE study [44, 45] and subsequently, after publication of the much awaited renal data from ONTARGET [46], it appeared that the risk/benefit ratio of RAAS combination therapy needed to be carefully reconsidered. While it has been pointed out that the ONTARGET study included only a relatively small number of CKD patients for its results to be applicable to the population of renal patients at large, especially those with overt proteinuria, this trial clearly showed that little o no CV benefit can be gained by combining ramipril and telmisartan in high risk patients. On the other hand, data show there is a price to pay with this therapeutic combination in terms of untoward effects, mainly hyperkalemia, hypotension and acute worsening of renal function, especially in the subgroup with impaired renal function.

The development of aliskiren [47] the first direct renin inhibitor (DRI), acting upstream of the enzymatic cascade and providing more profound inhibition as well as greater blood pressure lowering as compared to other agents, allowed to test the usefulness of higher degrees of RAAS suppression in the clinical setting. The AVOID study [48], conducted on 600 hypertensive patients with type 2 diabetes and overt renal disease, showed additional antiproteinuric action and stable renal function when aliskiren was given on top of losartan over a 6-month period, despite non significant changes in blood pressure.

More recently, however the strategy of combining aliskiren with an ACE-I or an ARB in diabetic patients at high cardiorenal risk was shown to entail potentially unfavorable effects. The ALTITUDE study, which was conducted on 8,500 patients, had to be prematurely stopped due to what turned out to be a lack of benefit, possibly associated with a greater risk of complications [15]. Thus, it appears that the risk-benefit ratio of pharmacological inhibition of the RAAS may vary along the renal continuum and seemingly paradoxical effects may be had when inhibition becomes too profound or the clinical setting becomes critical. While it is unlikely that dual RAAS inhibition does any good to cardiovascular health, further studies are certainly needed before it can be concluded that it is detrimental to CKD patients, and some ongoing trials may provide the much needed information. The VA NEPHRON-D trial will investigate the effect of combining losartan and lisinopril as compared to losartan alone in patients with diabetes and overt proteinuria [49]. Furthermore, the LIRICO [50] and VALID [51] studies will again evaluate dual therapy with either an ACE-I or an ARB in patients with micro-macroalbuminuria and in those with type 2 diabetes and overt nephropathy, respectively.

### Single Agent High Dose RAAS Inhibition

Based on experimental studies indicating different degrees of RAAS activity and drug effectiveness at the circulating and tissue level [52–54], with the latter possibly more accurately reflecting the development of organ damage in the long term, it has been proposed that supra pharmacological doses of RAAS-inhibiting drugs might provide greater renal benefit. Indeed, a few short-term studies have documented dose-dependent reductions of proteinuria, even regardless of hemodynamic changes [55–58]. Two studies have investigated this possibility under chronic conditions over the medium-long term. In the DROP study [57], treatment of patients with type 2 diabetes and albuminuria with increasingly high doses of valsartan (up to 640 mg/day) were associated with a significantly greater reduction in proteinuria and marginally lower blood pressure. Along the same lines, in the SMART study [58], 269 non diabetic CKD patients with clinical proteinuria were treated with candesartan up to 128 mg/day. While supramaximal doses did not seem to affect blood pressure values, urinary loss of protein was markedly reduced in a dose dependent fashion. Finally, in the ROAD study [59] up-titration of a single agent (benazepril or losartan) was compared to standard dose in 360 non diabetic patients with overt proteinuria over a three year follow-up period. Again, high dose treatment with each agent was associated with a greater antiproteinuric effect as compared to standard dose despite similar blood pressure reduction. Undoubtedly, high dose monotherapy with ACE-I or ARB appears to be a promising way to increase renal protection in CKD patients and one which certainly warrants a more in-depth evaluation by means of large well designed studies in the future.

### Aldosterone Receptor Antagonists

Several relatively small and short term studies have shown that both spironolactone and eplerenone can produce further reductions in albuminuria/proteinuria when added to optimal treatment with an ACE-I or an ARB [60]. These favorable results are corroborated by the results of at least two meta-analyses [61, 62]. However, no long term effect on renal function or survival has been documented, while treatment is associated with a greater risk of potentially dangerous untoward effects such as hyperkalemia, particularly when eGFR is below 30 ml/min. In one study, the addition of spironolactone 25 mg on top of high dose lisinopril (i.e., 80 mg) was associated with further proteinuria reduction regardless of blood pressure changes [63]. Thus, notwithstanding encouraging preliminary evidence, the routine use of an aldosterone receptor antagonist (ARA) in association with an ACE or an ARB appears to be premature and warrants further study.

## What Role Could There Be for RAAS Inhibition in the Prevention of Renal Damage?

The high CV and renal residual risk observed in patients with overt CKD despite multifactorial intensive treatment has prompted many to advocate prevention of renal damage in the subgroups of patients at risk as a way to more effectively fight the current epidemic of ESRD and associated CV diseases. Again, even in the field of primary prevention it has sometimes been difficult to distinguish the effect of blood pressure lowering from that of RAAS inhibition per se.

The BENEDICT study, carried out on patients with type 2 diabetes and hypertension, investigated the effectiveness of the ACE-inhibitor trandolapril as compared to the non-dihydropyridine calcium channel blocker verapamil given alone or in combination, over a mean 3.6 year follow-up period [64]. Treatment with trandolapril was associated with a significantly lower incidence of microalbuminuria, regardless of blood pressure values [65]. In the ADVANCE trial, the combination of perindopril-indapamide proved superior to placebo in preventing or delaying the onset of microalbuminuria, although lower blood pressure values were achieved in the active treatment arm [66]. Moreover, in the DIRECT study, a large trial carried out on mostly normotensive patients with type 1 and 2 diabetes, Candesartan did not produce any valuable effect in preventing the onset of albuminuria [67]. In another study, carried out on a smaller number of patients with type 1 diabetes, neither enalapril nor losartan showed any difference as compared to placebo in preventing microalbuminuria over a 5 year follow-up [68].

Finally, in the ROADMAP study, treatment with olmesartan was more effective than placebo in delaying the onset of microalbuminuria in a large cohort of type 2 diabetics, but again in the context of a slightly greater blood pressure lowering effect as compared to placebo [69].

Taken together, these data suggest a mildly favorable role for RAAS inhibitors in the prevention of renal damage in patients with diabetes, especially in the context of hypertension. This renal protective effect however, seems at least in part due to the blood pressure lowering effect of these agents.

## Conclusions

Achieving optimal blood pressure values through effective pharmacological treatment is associated with a reduction in proteinuria and a slower rate of renal deterioration over time in patients with CKD. RAAS-Is represent the antihypertensive drugs of choice and have been shown to provide greater antiproteinuric effects at comparable blood pressure regimens. Although only a few trials have directly compared ACE-Is to ARBs, these two classes of antihypertensives appear to have similar renal protective effectiveness. The latter however, are characterized by undisputed greater tolerability. Dual RAAS inhibition with an ACE-I/ARB combination does not provide additional CV benefits and might have serious side effects in the renal patient. Whether these risks are worth taking in selected cases, and if the slightly greater proteinuria reduction reported with this combination will actually translate into long term benefits on hard renal end point remains to be seen. There are currently insufficient data to recommend other RAAS combinations (with ARA or DRI) and high dose monotherapy with ACE-I or ARB in order to achieve greater renal protection. This is certainly an area that warrants further clinical investigation.

In the presence of CKD, achieving blood pressure targets almost invariably requires the association of several drugs [70], not rarely three or more. The type and dose or combination of diuretic(s) is often a key factor for therapeutic success [71]. Whenever a two-drug combination is sufficient to reach the desired blood pressure target, and in the absence of other compelling indication to the use of specific drug classes, RAAS-I and CCB can successfully be used as initial treatment [72]. Finally, clinicians should always be mindful that CKD requires all antihypertensive regimens to be tailored to each specific clinical situation and within the greater context of a multifactorial strategy which must include a healthy lifestyle and dietary habits. In particular, a decrease in sodium and phosphorus intake, and a combination of antiplatelet, lipid lowering drugs and optimal gluco-metabolic control as needed.
